# Novel plasma peptide markers involved in the pathology of CKD identified using mass spectrometric approach

**DOI:** 10.1007/s00109-019-01823-8

**Published:** 2019-08-05

**Authors:** Prathibha R. Gajjala, Heike Bruck, Heidi Noels, Georg Heinze, Francesco Ceccarelli, Andreas Kribben, Julio Saez-Rodriguez, Nikolaus Marx, Walter Zidek, Joachim Jankowski, Vera Jankowski

**Affiliations:** 1 Institute of Molecular Cardiovascular Research, University Hospital RWTH Aachen, Aachen, Germany; 2 Department of Internal Medicine, Nephrology, Rheumatology, Diabetology and Endocrinology, Helios Hospital Krefeld, Krefeld, Germany; 3 Section for Clinical Biometrics, Center for Medical Statistics, Informatics and Intelligent Systems, Medical University of Vienna, Vienna, Austria; 4 Joint Research Centre for Computational Biomedicine, RWTH Aachen University, Aachen, Germany; 5 Department of Nephrology, University Hospital Essen, University of Duisburg-Essen, Essen, Germany; 6 Institute for Computational Biomedicine, Faculty of Medicine Bioquant, Heidelberg University, Heidelberg, Germany; 7 Department of Internal Medicine I, University Hospital Aachen, Aachen, Germany; 8 Charité-Universitätsmedizin Berlin, Berlin, Germany; 9 Experimental Vascular Pathology, Cardiovascular Research Institute Maastricht (CARIM), University of Maastricht, Maastricht, Netherlands

**Keywords:** Chronic kidney disease, End-stage renal disease, Disease progression, Systems medicine, proteomics, peptidomics

## Abstract

**Abstract:**

Chronic kidney disease (CKD) may progress to end-stage renal disease (ESRD) at different pace. Early markers of disease progression could facilitate and improve patient management. However, conventional blood and urine chemistry have proven unable to predict the progression of disease at early stages. Therefore, we performed untargeted plasma peptidome analysis to select the peptides involved in progression, which are suitable for long prospective studies in future. The study consists of non-CKD (*n* = 66) and CKD (*n* = 106) patients with different stages. We performed plasma peptidomics on these subjects using chromatography and mass spectrometric approaches. Initially, we performed LC-ESI-MS and applied least absolute shrinkage and selection operator logistic regressions to select the peptides that are differentially expressed and we generated a peptidomic score for each subject. Later, we identified and sequenced the peptides with MALDI-MS/MS and also performed univariate and multivariate analyses with the clinical variables and peptidomic score to reveal their association with progression of renal disease. A logistic regression model selected 14 substances showing different concentrations according to renal function, of which seven substances were most likely occur in CKD patients. The peptidomic model had a global *P* value of < 0.01 with *R*^2^ of 0.466, and the area under the curve was 0.87 (95% CI, 0.8149–0.9186; *P* < 0.0001). The predicted score was significantly higher in CKD than in non-CKD patients (2.539 ± 0.2637 vs − 0.9382 ± 0.1691). The model was also able to predict stages of CKD: the Spearman correlation coefficient of the linear predictor with CKD stages was 0.83 with concordance indices of 0.899 (95% CI 0.863–0.927). In univariate analysis, the most consistent association of peptidomic score in CKD patients was with C-reactive protein, sodium level, and uric acid, which are unanticipated substances. Peptidomic analysis enabled to list some unanticipated substances that have not been extensively studied in the context of CKD but were associated with CKD progression, thus revealing interesting candidate markers or mediators of CKD of potential use in CKD progression management.

**Key messages:**

• Conventional blood and urine chemistry have proven unable to predict the progression of disease at early stages of chronic kidney disease (CKD).

• We performed untargeted plasma peptidome analysis to select the peptides involved in progression.

• A logistic regression model selected 14 substances showing different concentrations according to renal function.

• These peptides are unanticipated substances that have not been extensively studied in the context of CKD but were associated with CKD progression, thus revealing markers or mediators of CKD of potential use in CKD progression management.

## Introduction

The progression of chronic kidney disease (CKD) irrespective of its origin markedly contributes to the high prevalence of end-stage renal disease (ESRD). Numerous mechanisms of disease progression have been identified such as arterial hypertension, hyperphosphatemia, secondary hyperparathyroidism, and proteinuria [1–3]. However, the progressive nature of CKD is still an area of intensive research. Recently, the hypothesis was put forward that certain uremic toxins could create a vicious circle of disease progression, with the accumulation of uremic toxins self-perpetuating the loss of renal function [4]. Mainly small uremic toxins have been implicated in this mechanism of disease so far, such as indoxyl sulphate, phenylacetic acid p-cresylsulphate, 5-methoxythryptophan, canavaninosuccinate, acetylcarnitine, tiglycarnitine, and taurine [5–8]. On the other hand, the large group of so-called middle molecules has not been intensively studied in this respect so far. However, especially the role of peptides for CKD progression may be of interest, as several of these peptides may specifically activate signaling pathways involved in fibrosis, apoptosis, and other cellular mechanisms of renal damage. The identification of peptides affecting CKD may not only help to understand CKD progression, but could also establish new markers of disease progression. Early markers of disease progression could facilitate the attempts to reverse the frequently relentless progression to end-stage renal disease. In addition, also those peptides that showed decreased plasma concentrations with decreasing renal function may be of interest. Potentially, the loss of their renal protective effects may contribute to progressive renal failure.

The value of the LC-MS technique as a tool for identifying biomolecules such as peptides or metabolites is shown by recent studies in different renal diseases [8–12]. Using a mass spectrometric approach, we identified both peptides accumulating and peptides showing reduced concentrations with decreasing renal function, as these peptides may be of interest for establishing new therapeutic targets and/or new markers of CKD progression.

## Materials and methods

### Study subjects

The study cohort consisted of 66 non-CKD patients, three patients with estimated glomerular filtration rate (eGFR) greater than 60 ml/min with proteinuria, 44 patients with CKD 3, 15 patients with CKD 4, 9 patients with CKD 5, and 35 patients on dialysis with CKD 5. These subjects were recruited from the University Hospital Essen (Germany), Department of Nephrology. Inclusion criteria were (a) patients suffering from CKD 3–5 (KDOQI guideline) with stable chronic renal disease, eGFR < 60 ml/min for more than 3 months, and pathologic urinary sediment, proteinuria, and/or pathologic biopsy; (b) patients on dialysis for more than 3 months; and (c) patients without history of kidney disease, eGFR > 60 ml/min, unremarkable urinary sediment, no proteinuria, and normal kidney sonography, thus considered as non-CKD patients. We excluded patients younger than 18 and older than 85 years, pregnant or breast-feeding women, and/or patients not signing the informed consent document. The study was approved by the local ethical committee of the University Hospital Essen, Germany (ethical vote 08–3817) and all study subjects gave written informed consent.

### Biochemical measurements

Plasma-EDTA was isolated from peripheral blood by spinning at 2500 g and stored at − 80 °C. For biochemical characterization, blood urea nitrogen (BUN), calcium, creatinine, C-reactive protein (CRP), fibrinogen, hematocrit (HCT), high-density lipoprotein (HDL), hemoglobin, low-density lipoprotein (LDL), phosphate, parathyroid hormone (PTH), sodium, triglycerides, uric acid, urinary albumin, and white blood cell count (WBC) were measured using standard autoanalyzer techniques by the hospital laboratory. eGFR was calculated using the MDRD formula [13].

### Sample preparation for untargeted peptidomics

For peptide analysis, plasma-EDTA samples were processed. Prior to analysis, the plasma samples were randomly distributed to reduce the sampling errors. Samples were thawed and centrifuged at 13,000*g* for 5 min at 4 °C to remove the denatured proteins. Equal volumes (300 μl) of plasma were aliquoted for all samples, followed by addition of an internal standard to each sample (1 μg of [Sar^1^, Thr^8^]-angiotensin II, Sigma-Aldrich, Taufkirchen, Germany). By using 19.6 μl of 70% perchloric acid (Merck, Darmstadt, Germany), high abundant proteins were denatured and vortexed for 30 s. The samples were centrifuged at 13,000*g* for 2 min at 4 °C and the resulting supernatants were transferred to a reaction tube. The supernatant was increased in pH to 9.0 by adding 19.6 μl of 15 M potassium hydroxide (Sigma-Aldrich, Taufkirchen, Germany) and vortexed for 30 s. Until the separation by high-pressure liquid chromatography (HPLC), these samples were stored at − 20 °C for at least 24 h. Later, the samples were thawed and centrifuged at 13,000*g* for 5 min at 4 °C. The supernatant was collected in a reaction tube, the volume was increased to 5 ml with 0.1% trifluoroacetic acid (Sigma-Aldrich, Taufkirchen, Germany), and the pH was adjusted between 6 and 7 using 25% hydrochloric acid (Sigma-Aldrich, Taufkirchen, Germany) or 10 M sodium hydroxide.

### Peptide fractionation by reversed-phase chromatography

The peptides extracted from the plasma samples were fractionated using a C18-“Chromolith”™-reversed-phase chromatographic column (100 mm × 4.6 mm; Merck, Germany) combined with a UV detector and sample collector. Prior to loading, the column was rinsed with 100% ethanol (Sigma-Aldrich, Taufkirchen, Germany) followed by 100% milliQ water with 0.1% trifluoroacetic acid. The samples were loaded on the C18 column using the “BioLogic DuoFlow” HPLC injecting system (BioRad, USA). The peptides were fractionated using 0.1% TFA in water (v/v) (Fisher Scientific, Pittsburgh, USA) as a polar solvent (A) and 80% ethanol in water (v/v) as a non-polar solvent (B) at a flow rate of 1 ml/min for 36.9 min. A stepwise gradient was run as follows: 0–5 min 0% B, 5.1–11.1 min 20% B, 11.2–17.3 min 40% B, 17.3–22.3 min 100% B, 22.3–27.4 min 100% A, 31.4–36.9 min 0% B. Peptide separation and elution were monitored with UV absorbance at *λ*_280_ nm and collected in a 12-min interval. Desalting of the samples was performed simultaneously along with the chromatographic peptide separation. The resulted fractions were pooled and concentrated using the freeze-drying technique (Thermo Fisher Scientific, Lanerwehe, Germany) and then stored at − 20 °C for further analysis.

### Plasma peptidome analysis by liquid chromatography online coupled to electrospray ionization mass spectrometry (LC-ESI-MS)

For untargeted plasma peptidomic analysis of the cohort, we used capillary-HPLC system (Agilent 1200, Agilent, Germany) interfaced with an electrospray ionizer and HCT mass spectrometer (Bruker-Daltonics, Germany). For separation of peptides, “C18 SB Zorbax” column (150 × 0.5 mm; 5 μm, Agilent Technologies, Germany) was used and with the help of HyStar software (Bruker-Daltonics, Germany) data was acquired and processed. For chromatography, 0.1% formic acid in water as eluent A and 100% acetonitrile (Thermo Fisher Scientific, Lanerwehe, Germany) with 0.1% formic acid as eluent B were used. The flow rate was maintained at 60 μl/min for 22.0 min and the column temperature was constantly maintained around 50 °C. The column was equilibrated by rinsing with 100% acetonitrile followed by 100% LC-MS-grade water. The freeze-dried samples were reconstituted with 50 μl of 0.1% formic acid in water (v/v) and 2 μl of the reconstituted sampled was injected into the column by the auto-sampler of the chromatographic system. A linear gradient was applied as follows: 0.0–2.0 min 0% mobile phase B, 2.0–10.0 min 0–30% mobile phase B, 10.0–13.5 min 30–100% mobile phase B, 13.5–15.5 min 100% mobile phase B, 15.5–16.0 min 100–0% mobile phase B, and 16.0–22.0 min 0% B. The mass analyzer was operated in positive ion mode with source temperature at 300 °C. The nebulizer gas was maintained at 20 psi and the dry gas flow was adjusted to 9 l/min respectively. For the detection of peptides, global mode was used with the accumulation time set to 200 ms. The mass spectrometer was tuned in wide-mode option on *m*/*z* 800, operated in enhanced mode with the scan range of 100–1500 *m*/*z*. All data were acquired and processed using Compass 1.3 Software (Bruker-Daltonics, Germany).

### Data pre-processing

**Fig. 1:**
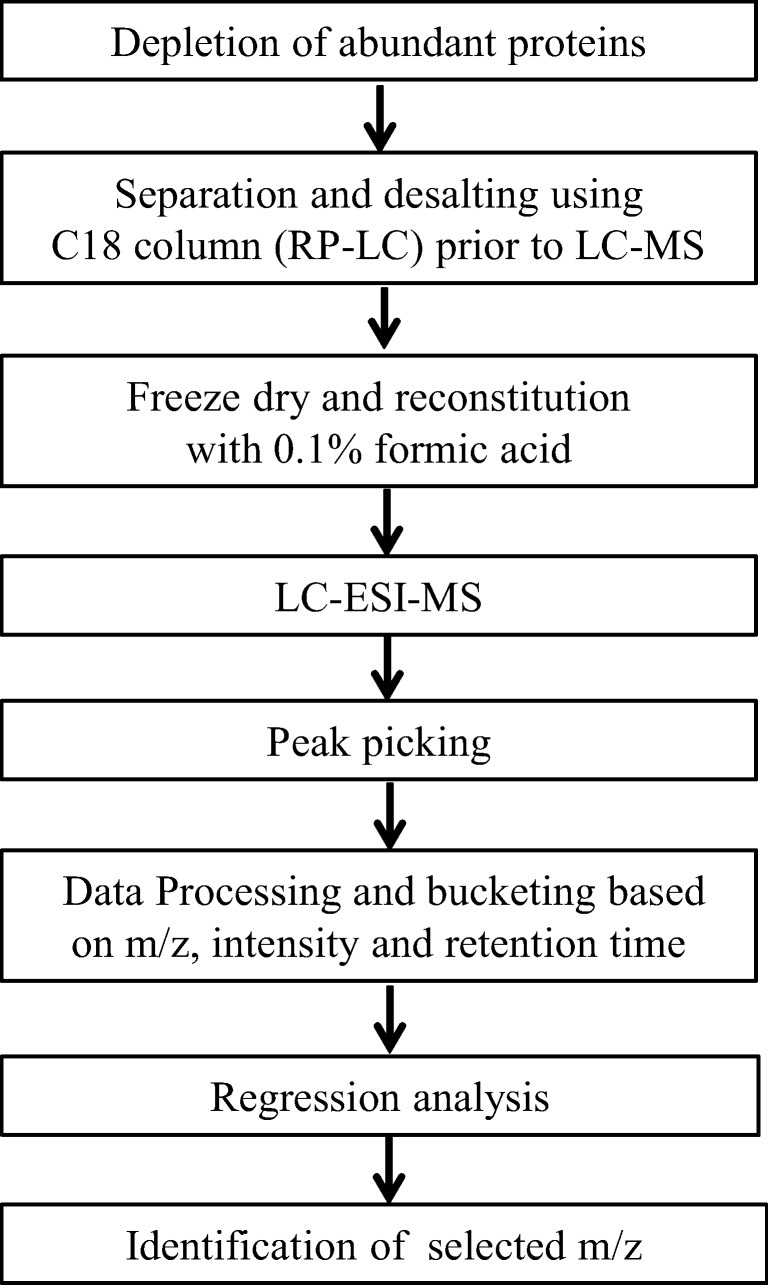
Outline of the steps followed in sample preparation for mass spectrometer and processing the data for statistical analysis

The raw data from LC-MS experiments were processed using “Data-analysis 4.0” (Bruker-Daltonics, Germany). For selecting high-quality peaks, so-called molecular features, the following parameters were used: (a) signal-to-noise ratio (*S*/*N*), 3; (b) correlation coefficient threshold, 0.7; (c) minimum compound length, 10 spectra; (d) smoothing width, 2. Spectral background subtraction was also performed. The data were transformed into buckets of 0.3 s difference in retention time and 0.2 *m*/*z* difference for statistical analysis using profile analysis software (Bruker-Daltonics, Germany) and were normalized with internal standard signal intensity. Then, an algorithm for peak picking (“find molecular features”) was employed to combine all ions that derive from the same compound, thus considerably reducing the size and complexity of the dataset to be analyzed. Further, to simplify the huge data for statistical analysis, chromatograms were transferred into buckets with the information on intensity, *m*/*z*, and the retention time of each molecular feature. Figure 1 a represents the workflow for selection of mass signals that distinguish CKD and non-CKD through a peptidomic-biostatistical integrated approach.

### Statistical methods

Statistical analysis was performed using “Statistical Analysis System” (SAS) software version 9.3 (SAS Institute, USA), R version 3.2.4. (Team 2016), and GraphPad Prism 6.0 software. Continuous variables were reported as means and standard deviation (SD) and compared between non-CKD patients and all stages of CKD patients using unpaired *t* tests. Categorical variables are reported as percentages and frequencies.

A plasma peptidomic classifier distinguishing CKD from non-CKD was developed using logistic and ridge regression. The peptide intensity values were transformed into log-base-2 values, and transformed values were used in all regression analysis. If peptide intensity was not detected by the mass spectrometer or a peptide was not present in a sample, it was scored as zero instead of a missing value. Firstly, we filtered out the peptides with more than 25% missing values across all samples. Later, zero values of a peptide were assigned to the minimum measured value of the respective peptide on the log scale minus a constant value of *d*. A logistic model was fitted with “least absolute shrinkage and selection operator” (LASSO) to select the peptides of importance by tuning a *λ* parameter such that the cross-validated deviance is minimized. Lastly, the model was re-estimated using ridge logistic regression based on two variables for each selected peptide: one dichotomous binary variable *D* that distinguished zero from non-zero measurements and a second continuous variable *X* which was equal to the logarithm of the mass-signal intensity of the peptide. If the mass-signal intensity was not detectable, it was set to the mean logarithm of peptide intensity to maintain interpretability of the binary variable’s coefficient. By tuning *d* by a leave-one-out cross-validation loop, we optimized the model. By this method, regression coefficients for detection/non-detection (*β*_*D*_) and for the peptide intensity (*β*_*X*_) were obtained and cross-validated linear predictor scores for each subject were calculated by repeating the whole model development process on datasets excluding that subject. The cross-validated linear predictor scores were compared between CKD stages and between non-CKD and CKD patients and their ability to distinguish between the two groups was described using ROC (*c*-) statistic. Pseudo *R*^2^ values were computed as the difference in predicted CKD probability between CKD and non-CKD patients (discrimination slope). [14]

Prior to assessing the associations between the cross-validated scores and clinical variables and to overcome the problem of sparse missing clinical data, we performed imputation of missing clinical variables using the R package MICE version 3.1 [15]. As imputation method, we selected predictive mean matching (PMM) and performed imputation on CKD and non-CKD samples separately. We then compared the distributions of the original and imputed data to make sure that the imputed values are indeed plausible values. To assess whether the cross-validated peptidomic scores were associated with clinical variables, we then performed the following statistical tests: Spearman’s correlation for continuous valued variables and two-sample *t* test for discrete binary valued variables, and multivariate logistic regression with CKD status (CKD vs non-CKD) as binomial outcome and the cross-validated peptidomic linear predictor score, hypertension status, hyperparathyroidism, age, sex, white blood cell count (WBC), waist, fibrinogen, hemoglobin, hematocrit, level of sodium, and uric acid as explanatory variables.

### Peptide identification using matrix-assisted laser desorption-time of flight mass spectrometry (MALDI-TOF-MS) and LTQ Orbitrap XL mass spectrometry

Peptides that were selected by the model were identified off-line from the reconstituted samples by MALDI-time of flight (TOF)/TOF fragment ion analysis as well as by LTQ Orbitrap XL mass spectrometry. One microliter of sample and α-4-hydroxycinnamic acid matrix was spotted on the target (MTP AnchorChip 400/384; Bruker-Daltonics, Germany) using dried droplet method α-4-hydroxycinnamic acid (Sigma-Aldrich, Germany) as described previously [12]. All measurements were performed on a Bruker Ultraflex-III TOF/TOF mass spectrometry (Bruker-Daltonics, Germany) that was equipped with a Smart Beam Laser operated at a repetition rate of 100–200 Hz and operated in positive mode. The instrument was calibrated to determine the calibration constants using standard peptides (Bradykinin (1–7), Ang II, P_14_R, ACTH (18–39); Sigma-Aldrich, Germany) resulting in an error of 100 ppm for the recorded mass spectra. The presented spectra are the representative average spectra with sum of 200 single-shot spectra for MS mode, and 600 for MS/MS mode. The positively charged ions mass spectra were analyzed in the reflector mode using delayed ion extraction. Using the LIFT option of the Ultraflex (Bruker-Daltonics, Germany), fragment ion spectra were recorded. Peptides were identified using the Mascot search engine (Matrix Science, UK) as well as by the RapideNovo 3.0.1 sequencing tool (Bruker-Daltonics, Germany) by searching for *Homo sapiens Proteins* based on the fragment ion-mass data.

To cross-check the identified molecular features, we performed mass spectrometric analyses using the high-end instrument MALDI LTQ Orbitrap XL (Thermo-Fisher Scientific, Germany) equipped with a nitrogen laser (MNL-100; LTB Lasertechnnik, Germany) operating at a wavelength of 337.1 nm with a spectral bandwidth of 0.1 nm, pulse repetition rate up to 60 Hz with 3-ns pulse width, and 75 μJ energy per pulse. Fourier transform mass spectrometric (FTMS) data were acquired in a measuring grid across the membrane area with a resolution of 60,000 in a positive range. The ion trap for the second scan event (MS/MS) and an activation type of “collision-induced dissociation” (CID) with a resolution of 60,000 in a positive range, a collision energy of 35 J, and an activation time of 30,000 ms. All data acquisitions were performed in the centroid mode using the mass range of 100–2000 *m*/*z*. The mass spectra were accumulated in with Xcaliber 2.1.0 and analyzed by Proteome Discoverer 1.4 (both Thermo Fischer Scientific, Germany). For identification, we downloaded the human.fast and uniprot.fast as well as an in-house Mascot database (Matrix Science Inc., US).

## Results

### Baseline characteristics of CKD and non-CKD patients

The study subjects were classified based on the eGFR and were divided into 106 CKD (cases) and 66 non-CKD (controls). The CKD and non-CKD patient’s characteristics are shown in Table 1. Thirty-two percent and 67.0% were males in non-CKD and CKD, respectively. CKD patients were older (67.5 (51.3–73.0) years) than non-CKD patients (53.0 (49.8–65.0) years). CKD patients have had significantly lower diastolic pressure (DBP, 71.91 ± 12.32 mm Hg) than non-CKD patients (75.45 ± 9.67 mm Hg). No significant differences were observed in height, weight, systolic pressure (SBP), and heart rate between these two groups during physical examination. Creatinine, BUN, CRP, fibrinogen, phosphate, PTH, uric acid, and WBC levels as well as urinary albumin were significantly higher and eGFR and HCT significantly lower in CKD patients. The lipid profiles were also significantly different between two groups. Lower hemoglobin levels were observed in CKD patients as anticipated.

**Table 1:** Baseline characteristics of the NT^CVD^ cohort

Variable	Non-CKD (*n* = 66)	CKD (*n* = 106)	*P* value
Demographics			
Age (years)*	53.0 (49.8–65.0)	67.5 (51.3–73.0)	0.0011
Male, *N* (%)	32 (48%)	67 (63%)	n.s
Physical examination			
DBP (mm Hg)	75.4 ± 9.7	71.9 ± 12.3	0.0301
Heart rate (bpm)*	67.0 (62.0–74.0)	69.0 (60.5–77.5)	n.s
Height (cm)	171.5 ± 9.5	171.9 ± 9.7	0.7479
SBP (mm Hg)	134.7 ± 19.8	138.9 ± 24.4	0.0945
Weight (kg)*	76.5 (66.8–89.3)	78.0 (69.0–91.9)	0.271
Biochemical data			
BUN (mg dL^−1^)*	15.0 (12.0–18.0)	32.0 (19.0–52.0)	< 0.0001
Calcium (mmol L^−1^)*	2.3 (2.3–2.4)	2.3 (2.2–2.4)	n.s
Creatinine (mg dL^−1^)*	1.0 (0.9–1.1)	2.8 (1.3–4.9)	< 0.0001
CRP (mg/L)*	0.5 (0.1–0.5)	0.5 (0.5–0.8)	0.0252
eGFR (ml/min/1.73 m^2^)*	68.5 (63.0–75.3)	28.5 (13.0–48.3)	< 0.0001
Fibrinogen (mg dL^−1^)*	334 (298–356)	410.5 (329.5–527.3)	< 0.0001
HCT	0.42 ± 0.003	0.39 ± 0.004	< 0.0001
HDL (mg dL^−1^)*	57.5 (46.0–70.0)	48.5 (39.0–60.0)	0.0007
Hemoglobin (g dL^−1^)*	13.8 (12.9–14.6)	12.6 (11.3–13.8)	< 0.0001
LDL (mg dL^−1^)*	127.5 (74.0–153.8)	102.0 (51.0–126.0)	< 0.0001
Phosphate (mg dL^−1^)*	3.4 (3.1–3.8)	3.7 (3.3–4.8)	0.0006
PTH (ng L^−1^)*	39.7 (33.5–55.1)	85.2 (44.8–138.1)	< 0.0001
Sodium (mmol L^−1^)	139.7 ± 0.24	138.9 ± 0.28	0.0448
Triglyceride (mg dL^−1^)*	101.0 (71.3–153.3)	121.0 (81.3–173.0)	0.0277
Uric acid (mg dL^−1^)*	5.4 (4.8–6.17)	5.9 (4.5–7.8)	0.0092
Urine albumin (mg L^−1^)	0	77.2 (3.2–380.4)	0.0246
WBC (10^9^ cells L^−1^)*	5.89 (4.9–6.9)	6.6 (5.4–8.2)	0.0025
Renal replacement therapy			
Dialysis (CKD5, in %)	0	33.0	

*Median and interquartile range. *BUN* blood urea nitrogen, *CRP* C-reactive protein, *DBP* diastolic blood pressure, *eGFR* estimated glomerular filtration rate, *HCT* hematocrit, *HDL* high-density lipoprotein, *LDL* low-density lipoprotein, PTH para thyroid hormone, *SBP* systolic blood pressure, *WBC* white blood cell, *n.s* not significant

### Plasma peptidomic analysis

**Fig. 2:**
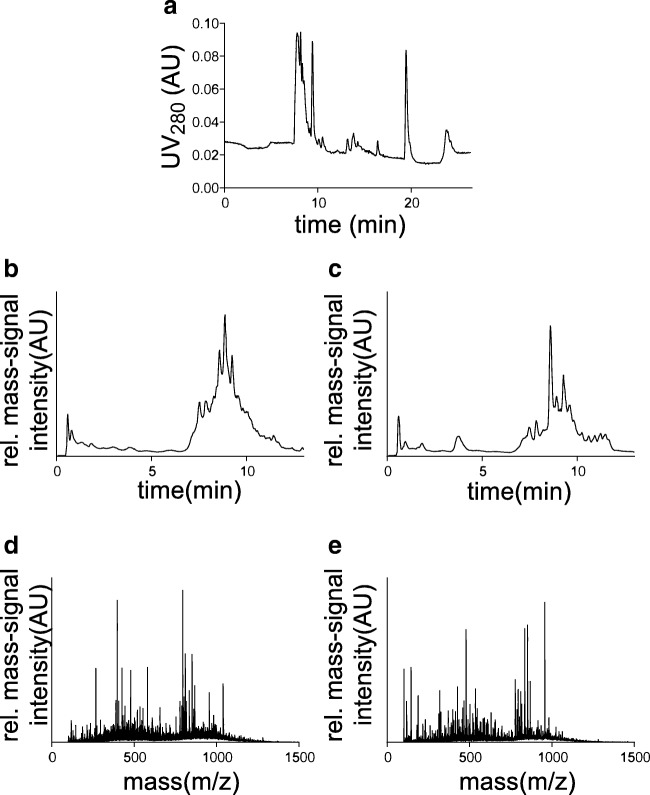
Analysis of the preprocessed samples using online coupled liquid chromatography electrospray ionization mass spectrometry. **a** Separation and desalting of peptides on reversed-phase chromatographic column performed using HPLC. Peptides are separated and eluted based on the hydrophobic nature of the solvent to reduce the sample complexity. The elution of peptides is monitored by the UV absorbance at *λ*_280_ nm and desalting is monitored by the conductometer. **b** Characteristic total ion chromatogram of a sample from the CKD group. **c** Characteristic total ion chromatogram of a sample from the non-CKD subject group. **d** Corresponding average mass spectrum of a sample from the CKD group. **e** Corresponding average mass spectrum of a sample from the non-CKD subject group. AU indicates arbitrary units

**Fig. 3:**
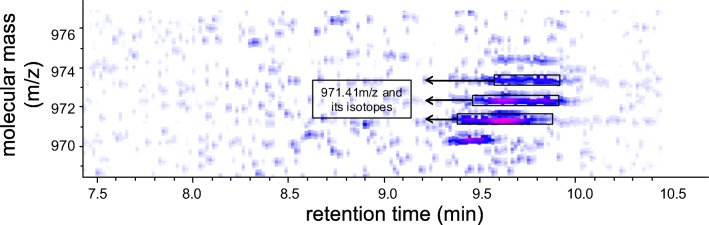
Density view of the 971.41 *m*/*z* selected feature by the model with their naturally occurring isotopic forms

The plasma samples were processed to enrich the naturally occurring endogenous peptides by depleting the high abundant plasma proteins by acid denaturation. The processed samples were later fractionated and desalted to reduce the complexity of the samples. Elution was monitored by UV absorbance at *λ*_280_ nm using analytical HPLC. Figure 2a shows a representative chromatogram of the separated peptides eluted with 80% ethanol. Figure 2b and c represent the characteristic total ion chromatogram of a CKD and a non-CKD patient respectively, demonstrating differences in their total ion chromatogram. Figure 2d and e represent the respective average spectra of a CKD and a non-CKD, which show significant differences in the mass signals. Figure 3 shows the density view of one of the selected features with their naturally occurring isotopes, which is detected through LC-ESI-MS.

### Performance of the model

The raw mass spectrometric profiles were processed, and then buckets with *m*/*z* and retention time were generated based on the difference in the retention time and *m*/*z* of all samples. The bucket table was analyzed to find the significant differences in molecular masses between non-CKD and CKD and a linear prediction model was developed to distinguish among controls (non-CKD) and cases (stages 2–5d of CKD). Firstly, the prediction model was developed using regression analysis. Fourteen molecular features were selected. The regression coefficients of the 14 features selected for the model are shown in Table 2. Later, we applied the same model building procedure to the features that were not selected in the first model, but no further features were selected.

**Table 2:** Selected features and their coefficients

*m*/*z*	Description of intensity values		Multivariable model		
	Proportion non-zero among progressors	Proportion non-zero among non-progressors	*β*_*X*_ (per doubling of intensity	*β*_*D*_ (detection vs non-detection of peptide)	Partial *R*^2^
Upregulated in CKD					
367.22	0.94	0.78	0.19	1.58	0.006
384.19	0.79	0.39	0.13	0.92	0.002
971.41	0.5	0.16	0.09	1.37	− 0.006
551.13	0.81	0.46	0.09	1.33	− 0.001
972.39	0.36	0.14	0.08	1.23	− 0.004
831.17	0.49	0.07	0.07	0.65	− 0.006
129.85	0.59	0.28	0.05	0.18	− 0.005
Downregulated in CKD					
188.42	0.86	0.96	− 0.16	− 1.13	0.003
537.08	0.5	0.68	− 0.14	− 0.7	0.011
433.26	0.51	0.83	− 0.11	− 0.68	0.003
389.27	0.27	0.62	− 0.1	− 0.83	0
342.25	0.41	0.7	− 0.05	− 0.58	− 0.001
636.36	0.32	0.62	− 0.02	− 0.67	− 0.002
576.05	0.66	0.22	− 0.01	0.32	− 0.007

**Fig. 4:**
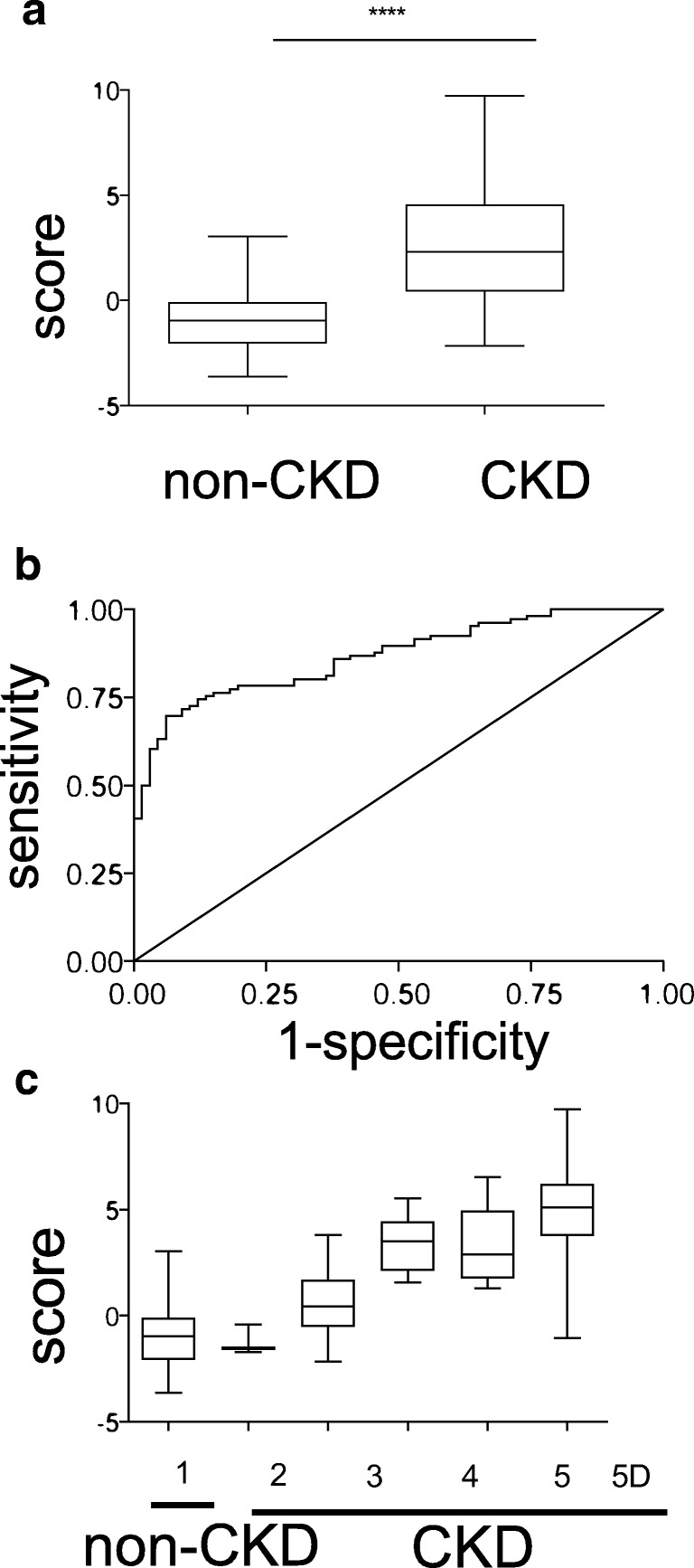
Development of a plasma peptidomic multivariable model using LASSO logistic and ridge regressions to distinguish the CKD and non-CKD patients. **a** Box plot of a cross-validated plasma peptidomic model for CKD, *P* < 0.0001. **b** Receiver operating characteristic curve of the cross-validated plasma peptidomic model with an area under the curve of 0.87 (95% confidence interval, 0.8149–0.9186; *P* < 0.0001). **c** Box plot depicting the cross-validated plasma peptidomic predictor with CKD stages as dependent variable. The higher the predictor score from these 14 features, the higher the stage of CKD. The number on *x*-axis represents the stage of CKD

The first peptidomic model had a global *P* value < 0.01 with an overall *R*^2^ of 0.466. Among the selected features, seven were downregulated and seven were upregulated in CKD compared with non-CKD. The predicted score was significantly higher in CKD (stages 2–5d) than in non-CKD patients (2.539 ± 0.2637 vs − 0.9382 ± 0.1691), as shown in the box plots of Fig. 4a. With the linear model, we conducted a leave-one-out cross-validation and generated hereby a cross-validated predictor. The diagnostic power of the 14 selected molecular features was measured using ROC analysis. The area under curve of the model was found to be 0.87 (95% confidence interval, 0.8149–0.9186; *P* < 0.0001) as shown in Fig. 4b**.**

Further, the CKD patients were subdivided into the respective stages of CKD (GFR > 60 ml/min with proteinuria, CKD stage 3, CKD stage 4, CKD stage 5, and patients on dialysis (CKD stage 5D)) with the unmodified cross-validated predictor as shown in Fig. 4c. We observed that the score generated for each subject was directly proportional to the stage of CKD **(**Fig. 4c**)**. The Spearman correlation coefficients of the linear predictor and the cross-validated linear predictor with the CKD stages were 0.83 and 0.78, respectively. The concordance indices were 0.899 (95% CI 0.863–0.927) for the linear predictor and 0.872 (0.830–0.905) for the cross-validated linear predictor.

### Clinical correlates of the peptidomic score

In correlation analyses, the peptidomic scores were most significantly correlated to C-reactive protein (*r* = 0.2, uncorrected *P* value = 0.03), sodium level (*r* = − 0.2, uncorrected *P* value = 0.03) and uric acid (*r* = − 0.21, *P* value = 0.02) (see Table 3 for all uncorrected *P* values). There was no significant difference in CKD patients due to sex (2.40 ± 2.53 vs 2.70 ± 2.93; *P* = 0.59) or dyslipoproteinemia (2.79 ± 2.11 vs 2.42 ± 2.82; *P* = 0.56).

**Table 3:** Correlation analysis of clinical variables with cross-validated peptidomic score in CKD patients

Variable	Correlation coeff	*P* value
Age	0.085	0.389
BNP	0.004	0.971
BUN	− 0.071	0.472
Calcium	− 0.061	0.535
Chloride	− 0.103	0.297
Cholesterol	0.087	0.377
Creatinine	− 0.001	0.996
CRP	0.203	0.039
DBP	− 0.033	0.741
eGFR	0.059	0.555
Fibrinogen	0.010	0.916
HbA_1_c	0.018	0.857
HDL	− 0.053	0.592
Heart rate	0.047	0.637
Height	− 0.154	0.119
Hematocrit	0.118	0.231
Hemoglobin	0.109	0.271
LDL	− 0.035	0.724
Lipoprotein A	− 0.069	0.485
Glucose	0.057	0.567
Phosphate	0.044	0.659
Potassium	0.063	0.528
PTH	− 0.056	0.574
SBP	0.011	0.909
Sodium	− 0.207	0.035
Total protein	0.047	0.634
Triglycerides	− 0.037	0.711
Troponin	0.049	0.616
Uric acid	− 0.216	0.028
Urinary albumin	0.154	0.119
Waist	− 0.047	0.634
WBC	0.045	0.651
Weight	0.002	0.986

Significance code in the table: *BNP* brain natriuretic peptide, *BUN* blood urea nitrogen, *CRP* c-reactive protein, *DBP* diastolic blood pressure, *eGFR* estimated glomerular filtration rate, *HbA*_*1*_*c* glycated hemoglobin, *HDL* high density lipoprotein, *LDL* low density lipoprotein, *PTH* parathyroid hormone, *SBP* systolic blood pressure, *WBC* white blood cell

In multiple logistic regression analysis, we first used LASSO logistic regression with 10-fold cross-validation to identify a solid sub-list of clinical features to include in the final multiple logistic regression model. LASSO was trained on a random subset (80%) of the samples and the most predictive features were chosen. We performed multivariable logistic regression with CKD vs non-CKD status as binomial outcome and the cross-validated peptidomic score, hypertension status, hyperparathyroidism, age, sex, waist, WBC, fibrinogen, hemoglobin, hematocrit, level of sodium, and uric acid as explanatory variables. The association with the presence of CKD was significant for the peptidomic score, hyperparathyreoidism, and hematocrit as shown in Table 4. We used the remaining 20% of samples to assess the ability of the model to distinguish between CKD and non-CKD patients. The model had an AUC = 0.95.

**Table 4:** Logistic regression model linking the presence of CKD with the peptidomic score and major clinical variables

Variables	Odds ratio	95% CI	*P* value
Peptidomic score	2.018	1.44–2.82	4.4e−05***
Hypertension (yes)	2.076	0.42–10.19	0.36814
Hyperparathyreoidism (yes)	15.91	2.79–90.80	0.00184**
Age (per year)	1.01	0.96–1.06	0.68837
Sex (female)	2.049	0.45–9.23	0.35019
WBC (10^9^ cells L^−1^)	0.895	0.59–1.35	0.60140
Hemoglobin (g/dL)	0.925	0.64–1.34	0.67714
Hematocrit	< 0.001	0–0.57	0.04366*
Fibrinogen (mg dL^−1^)	1.006	0.99–1.01	0.19046
Sodium (mmol L^−1^)	1.05	0.80–1.37	0.72399
Uric acid (mg dL^−1^)	1.339	0.89–2.00	0.15889
Waist (cm)	1.039	0.98–1.09	0.14602

Significance code in the table: ‘***’< 0.001, ‘**’< 0.01, ‘*’< 0.05, ‘.’< 0.1

### Identification and sequencing of selected features

**Fig. 5:**
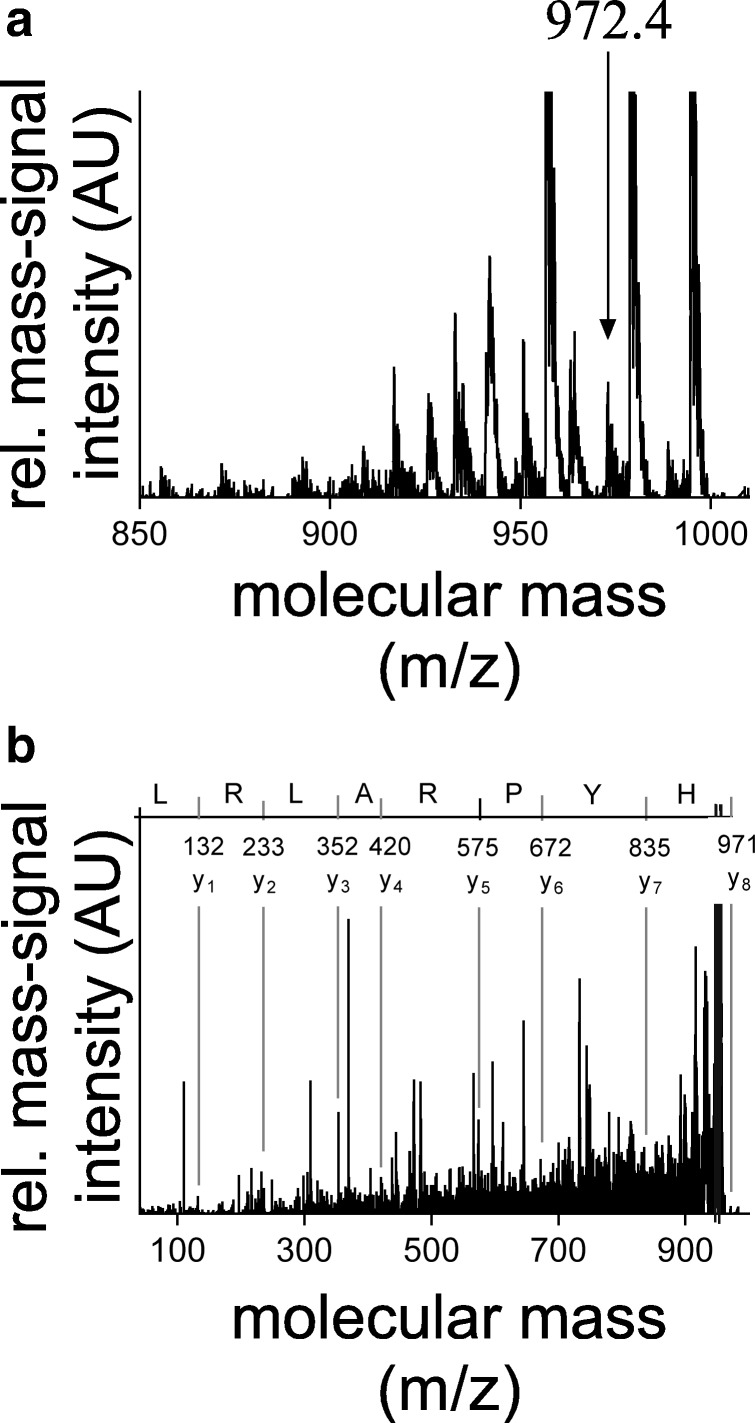
Identification of selected features by MALDI TOF/TOF that distinguish the CKD and non-CKD patients. **a** Representative mass spectrum of the selected molecular feature with *m*/*z* of 972.4. **b** Representative fragmentation spectra of the selected molecular feature with *m*/*z* of 972.4 with amino acid sequence HYPRALCL. AU indicates arbitrary units

After selection of molecular features, their molecular structures were identified using MALDI TOF/TOF-MS and LTQ Orbitrap XL. Figure 5a represents the MS spectrum of a feature with 972.4 *m*/*z*, which is upregulated in CKD patients and Fig. 5b represents its respective MS/MS fragmentation ion spectra, which is a fragment of amiloride-sensitive amine oxidase (AOC1, ABP1, DAO1) with the amino acid sequence of HYPRALCL. Also, fragments of osteocalcin (BGLAP), angio-associated migratory cell protein (AAMP), putative inactivation escape (INE1), sodium bicarbonate transporter like protein (SLC4A11/BTR1), and lysine were also upregulated in CKD. Fragments of erythrocyte membrane glycopeptide, thymosin beta-10 (TMSB10/PTMB10/THYB10), humanin (MT-RNR2/HN), aldehyde dehydrogenase family 3 member 1 (ALDH3A1/ALDH3), and tryptophan were identified protein fragments that were downregulated in CKD patients. Further, the model selected osteocalcin based on three peptides with different amino acid sequences, and AOC1 based on two peptides with similar sequence. The list of identified features is shown with their sequences in Table 5. Afterwards, literature mining was performed on the identified peptides to investigate their pathophysiological roles. Although, few of the proteins were reported in the context of renal failure, most of them were related to either atherosclerosis, hypertension, or cardiovascular diseases. Each protein role is discussed in detail in the “Discussion” section.

**Table 5:** List of the peptide sequences and their respective proteins identified by MALDI-MS

RT min/*m*/*z*	Sequence	Identification
9.3/129.85	K	Lysine
3.8/188.42	W	Tryptophan
2.1/342.25	LNP	Osteocalcin
1.8/367.22	GYE	Angio-associated migratory cell protein (AAMP)
6.9/384.19	DHI	Osteocalcin
9.2/389.27	LKK	Thymosin beta-10 (TMSB10/PTMB10/THYB10)
9.7/433.26	LLTS	Humanin (MT-RNR2/HN)
1.4/537.08	HDHGA	Erythrocyte membrane glycopeptide (BGLAP)
8.2/551.13	GLPQH	Putative inactivation escape (INE1)
6.9/576.05	CELNP	Osteocalcin
12.4/636.36	TQTSSGG	Aldehyde dehydrogenase family 3 member 1 (ALDH3A1/ALDH3)
8.1/831.17	ALFSGQPL	Sodium bicarbonate transporter like protein (SLC4A11/BTR1)
9.6/971.41	HYPRALCL	Amiloride-sensitive amine oxidase (AOC1, ABP1, DAO1)
9.8/972.39	HYPRALCL	Amiloride-sensitive amine oxidase

## Discussion

Peptide-based comparative analyses of non-CKD and CKD patients at different stages were performed using a peptido-biostatistic integrated approach on human plasma. As plasma provides a snapshot of the molecular status of the CKD patients at the point of collection, any alterations in the blood peptidome in CKD can be detected by comparing with non-CKD patients. Thus, we developed a plasma peptidomic linear predictor model to determine CKD stages based on the significant differences between CKD and non-CKD. The predictor model developed discriminates between CKD and non-CKD and has great stability, as no other features were identified after excluding the selected features at the time of the model development process. The little drop in the *R*^2^ of each selected feature illustrates that it is not a single feature that influences the model, but rather a panel of features are essential for the superior performance of the model. The model also differentiates between different stages of CKD based on selected features score, as shown in Fig. 4c.

A plethora of molecular mechanisms/pathways have been illustrated to be involved in CKD pathology, such as vascular calcification and stiffness due to an imbalance in calcium and phosphate levels [16], the renin-angiotensin-aldosterone system (RAAS) [17], endothelial dysfunction [18], inflammation [19], coagulation processes [20], and oxidative and metabolic stress [21, 22]. The results of this study show that in CKD patients, several peptides show increased plasma concentrations, whereas others are lower than in non-CKD condition. This finding indicates that the changes in plasma peptide concentrations in renal failure are not simply due to accumulation as a consequence of diminished urinary excretion. In addition to lower excretion, also up- and downregulation of peptide synthesis may play a role. Furthermore, uremic toxins may inhibit or activate peptidases involved in the generation and/or degradation of these peptides.

Among those peptides showing increased concentrations in renal failure patients, several substances deserve mention: “angio-associated migratory cell protein” (AAMP) plays a role in angiogenesis and cellular adhesion. Blocking AAMP inhibits the formation of neo-intima in advanced atherosclerosis by driving the proliferation and migration of smooth muscle cells [23]. The latter may explain why patients with CKD have a poor clinical outcome after percutaneous coronary intervention [24]. Further, although these findings do not explain the role of increased AAMP levels in renal failure, it may be speculated that AAMP is reactively stimulated.

“Amiloride-sensitive amine oxidase” has been identified as a key enzyme in renal fibrosis, which is one important histopathological correlate of renal disease progression [25]. Indeed, Lin et al. found a significant association between diamine oxidase activity and renal disease progression [26]. The present results suggest that also plasma diamine oxidase levels may serve as a marker of renal disease progression. Moreover, if renal interstitial fibrosis can be influenced by modifying diamine oxidase activity, diamine oxidase may also emerge as a promising therapeutic target to slow progression of renal disease.

The “sodium bicarbonate transporter like protein” (Cl/HCO_3_) is a ubiquitous cellular transport system. It is especially highly expressed in the cornea and in renal tissue. Cl/HCO_3_ transporter knockout mice show polyuria and low urinary osmolality [27]. In contrast to its denomination, this transporter may act as a NH_3_/2H^+^ cotransporter [28]. The increased levels in renal failure may be part of a counter regulation to maintain acid-base homeostasis under these conditions.

Several peptide concentrations were found to be decreased in CKD; “Thymosin β 10” is a peptide inhibiting angiogenesis [29]. Suppression of thymosin β 10 increases cell migration [30]. Currently it is difficult to integrate this finding in the pathophysiology of renal failure. Embryonic kidneys express thymosin β 10 especially in the proximal and distal tubules [31], but it remains open whether reduced thymosin β 10 plasma levels may reflect altered regenerative processes in renal tissue.

“Humanin” is a mitochondrial peptide showing protective effects in ischemia/reperfusion injury by decreasing reactive oxygen species production [32, 33]. Moreover, it was shown that humanin increases glucose-stimulated insulin secretion [34]. Humanin also exhibits anti-inflammatory effects in hypercholesterolemic apoE-deficient mice [35], improves endothelial function and inhibits atherogenesis [36]. This spectrum of actions suggests that decreased humanin production may be related to the pathophysiology of uremia characterized by insulin insensitivity, activated inflammatory processes, increased oxidative stress, impaired endothelial function, and premature atherosclerosis.

*“*Aldehyde dehydrogenase family 3 member 1” (ALDH3A1) serves as a catalyzer for oxidizing several aldehydes thereby inducing their detoxification, thus helping to maintain cellular homeostasis under the conditions of oxidative stress [37]. Further, ALDH3A1 also has anti-oxidant capacities through the generation of the anti-oxidant NADPH and by scavenging ROS [38]. Moreover, ALDH3A1 serves as a chaperone [39]. Therefore, decreased levels of ALDH3A1 could be a further important factor in uremic pathophysiology, both with respect to the increased oxidative stress in uremia and to impaired protein function.

Apart from proteins, the model includes two free deaminated forms of amino acids, lysine and tryptophan; the latter was downregulated in CKD patients. Exogenous supplementation of lysine was reported to normalize/reduce blood pressure in hypertensive patients [40], osteoporosis, and incidence of cardiovascular disease [41]. However, its role in renal failure seems to be protective due to increase in lysine levels. This feature was found to be upregulated and clearly shows the importance of this feature to be included in the biostatistical model.

The present study is an initial step to select plasma peptides as potential biomarkers of renal disease progression. In contrast to earlier studies on plasma proteomics/peptidomics, this study uses a non-selective (untargeted) approach [42]. No selection of peptides/proteins of interest was done with respect to the number or identity of analyzed proteins and peptides. A selective approach based on our current understanding of uremic pathophysiology carries the risk that important peptides involved in important mechanisms of uremic pathophysiology, but yet unidentified, may be excluded from analysis.

The main strength of the study is that a rigorous cross-validation was performed to avoid overestimation of the model. However, as drawback of this peptidomic study, the resulting data do not inform on a potential causal relationship between the identified peptides and CKD. Validation of the model in other cohorts would provide further support for a potential role of the identified peptides as biomarker and/or mediator of CKD, after which functional and mechanistic analyses might pave a path towards identifying novel culprits of CKD progression and thus promising drug targets. In this study, we used patients from different etiologies since CKD itself is a complex disease. The candidate peptides selected in this study fulfilled the criteria that (1) they showed plasma concentrations significantly different from those in non-CKD subjects and (2) their plasma concentrations are related to the stage of renal insufficiency. In a next step, cohorts with prospective clinical data will have to be analyzed to assess whether one or several of these peptides may represent a biomarker predictive of renal disease progression.

In conclusion, peptide statistical analysis of plasma of CKD patients enabled us to list possible biomarkers and/or mediators of CKD, with plasma levels altered dependent on CKD stage. Thus, a peptidomic platform as in this study may be beneficial along with other advancing laboratory technology in routine clinical practice for stratification of CKD and could also facilitate or/and improve CKD progression management.

## References

[1] Jafar TH, Stark PC, Schmid CH, Landa M, Maschio G, de Jong PE, de Zeeuw D, Shahinfar S, Toto R, Levey AS, for the AIPRD Study Group* (2003). Progression of chronic kidney disease: the role of blood pressure control, proteinuria, and angiotensin-converting enzyme inhibition: a patient-level meta-analysis. Ann Intern Med.

[2] Daniels CE, Lasky JA, Limper AH, Mieras K, Gabor E, Schroeder DR, Imatinib IPFSI (2010). Imatinib treatment for idiopathic pulmonary fibrosis: randomized placebo-controlled trial results. Am J Respir Crit Care Med.

[3] Schlondorff DO (2008). Overview of factors contributing to the pathophysiology of progressive renal disease. Kidney Int.

[4] Liabeuf S, Cheddani L, Massy ZA (2018) Uremic toxins and clinical outcomes: the impact of kidney transplantation. Toxins (Basel) 10. 10.3390/toxins10060229 10.3390/toxins10060229 PMC6024850 29874852

[5] Richeldi L, du Bois RM, Raghu G, Azuma A, Brown KK, Costabel U, Cottin V, Flaherty KR, Hansell DM, Inoue Y, Kim DS, Kolb M, Nicholson AG, Noble PW, Selman M, Taniguchi H, Brun M, le Maulf F, Girard M, Stowasser S, Schlenker-Herceg R, Disse B, Collard HR (2014). Efficacy and safety of nintedanib in idiopathic pulmonary fibrosis. N Engl J Med.

[6] Jankowski J, van der Giet M, Jankowski V, Schmidt S, Hemeier M, Mahn B, Giebing G, Tölle M, Luftmann H, Schlüter H, Zidek W, Tepel M (2003). Increased plasma phenylacetic acid in patients with end-stage renal failure inhibits iNOS expression. J Clin Invest.

[7] Fujii H, Goto S, Fukagawa M (2018) Role of uremic toxins for kidney, cardiovascular, and bone dysfunction. Toxins (Basel) 10. 10.3390/toxins10050202 10.3390/toxins10050202 PMC5983258 29772660

[8] Chen DQ, Cao G, Chen H, Argyopoulos CP, Yu H, Su W, Chen L, Samuels DC, Zhuang S, Bayliss GP, Zhao S, Yu XY, Vaziri ND, Wang M, Liu D, Mao JR, Ma SX, Zhao J, Zhang Y, Shang YQ, Kang H, Ye F, Cheng XH, Li XR, Zhang L, Meng MX, Guo Y, Zhao YY (2019). Identification of serum metabolites associating with chronic kidney disease progression and anti-fibrotic effect of 5-methoxytryptophan. Nat Commun.

[9] Zhao YY, Lint RC (2014). Metabolomics in nephrotoxicity. Adv Clin Chem.

[10] van den Broek I, van Dongen WD (2015). LC-MS-based quantification of intact proteins: perspective for clinical and bioanalytical applications. Bioanalysis.

[11] Zhao YY, Wang HL, Cheng XL, Wei F, Bai X, Lin RC, Vaziri ND (2015). Metabolomics analysis reveals the association between lipid abnormalities and oxidative stress, inflammation, fibrosis, and Nrf2 dysfunction in aristolochic acid-induced nephropathy. Sci Rep.

[12] Gajjala PR, Jankowski V, Heinze G, Bilo G, Zanchetti A, Noels H, Liehn E, Perco P, Schulz A, Delles C, Kork F, Biessen E, Narkiewicz K, Kawecka-Jaszcz K, Floege J, Soranna D, Zidek W, Jankowski J (2017). Proteomic-biostatistic integrated approach for finding the underlying molecular determinants of hypertension in human plasma. Hypertension.

[13] Levey AS, Coresh J, Greene T, Marsh J, Stevens LA, Kusek JW, Van Lente F, Chronic Kidney Disease Epidemiology C (2007). Expressing the Modification of Diet in Renal Disease Study equation for estimating glomerular filtration rate with standardized serum creatinine values. Clin Chem.

[14] Tjur T (2009). Coefficients of determination in logistic regression models—a new proposal: the coefficient of discrimination. Am Stat.

[15] Stef van Buuren KG-O (2011). Mice: multivariate imputation by chained equations in R. J Stat Softw.

[16] Smith ER (2016). Vascular calcification in uremia: new-age concepts about an old-age problem. Methods Mol Biol.

[17] Gajjala PR, Sanati M, Jankowski J (2015). Cellular and molecular mechanisms of chronic kidney disease with diabetes mellitus and cardiovascular diseases as its comorbidities. Front Immunol.

[18] Luczak M, Formanowicz D, Marczak L, Pawliczak E, Wanic-Kossowska M, Figlerowicz M, Stobiecki M (2015). Deeper insight into chronic kidney disease-related atherosclerosis: comparative proteomic studies of blood plasma using 2DE and mass spectrometry. J Transl Med.

[19] Hwang IC, Park HE, Kim HL, Kim HM, Park JB, Yoon YE, Lee SP, Kim HK, Cho GY, Sohn DW, Kim YJ (2016). Systemic inflammation is associated with coronary artery calcification and all-cause mortality in chronic kidney disease. Circ J.

[20] Suarez-Alvarez B, Liapis H, Anders HJ (2016). Links between coagulation, inflammation, regeneration, and fibrosis in kidney pathology. Lab Investig.

[21] Gamboa JL, Billings FT, Bojanowski MT, Gilliam LA, Yu C, Roshanravan B, Roberts LJ, Himmelfarb J, Ikizler TA, Brown NJ (2016). Mitochondrial dysfunction and oxidative stress in patients with chronic kidney disease. Physiol Rep.

[22] Fadaee SB, Beetham KS, Howden EJ, Stanton T, Isbel NM, Coombes JS (2017). Oxidative stress is associated with decreased heart rate variability in patients with chronic kidney disease. Redox Rep.

[23] Vogt F, Zernecke A, Beckner M, Krott N, Bosserhoff AK, Hoffmann R, Zandvoort MA, Jahnke T, Kelm M, Weber C (2008). Blockade of angio-associated migratory cell protein inhibits smooth muscle cell migration and neointima formation in accelerated atherosclerosis. J Am Coll Cardiol.

[24] Gruberg L, Dangas G, Mehran R, Mintz GS, Kent KM, Pichard AD, Satler LF, Lansky AJ, Stone GW, Leon MB (2002). Clinical outcome following percutaneous coronary interventions in patients with chronic renal failure. Catheter Cardiovasc Interv.

[25] Wong MY, Saad S, Pollock C, Wong MG (2013). Semicarbazide-sensitive amine oxidase and kidney disease. Am J Physiol Renal Physiol.

[26] Lin MS, Li HY, Wei JN, Lin CH, Smith DJ, Vainio J, Shih SR, Chen YH, Lin LC, Kao HL, Chuang LM, Chen MF (2008). Serum vascular adhesion protein-1 is higher in subjects with early stages of chronic kidney disease. Clin Biochem.

[27] Groger N, Frohlich H, Maier H, Olbrich A, Kostin S, Braun T, Boettger T (2010). SLC4A11 prevents osmotic imbalance leading to corneal endothelial dystrophy, deafness, and polyuria. J Biol Chem.

[28] Zhang W, Ogando DG, Bonanno JA, Obukhov AG (2015). Human SLC4A11 is a novel NH3/H+ co-transporter. J Biol Chem.

[29] Lee SH, Son MJ, Oh SH, Rho SB, Park K, Kim YJ, Park MS, Lee JH (2005). Thymosin {beta}(10) inhibits angiogenesis and tumor growth by interfering with Ras function. Cancer Res.

[30] Sribenja S, Wongkham S, Wongkham C, Yao Q, Chen C (2013). Roles and mechanisms of beta-thymosins in cell migration and cancer metastasis: an update. Cancer Investig.

[31] Gerosa C, Fanni D, Nemolato S, Locci A, Marinelli V, Cabras T, Messana I, Castagnola M, Monga G, Fanos V, Faa G (2010). Thymosin beta-10 expression in developing human kidney. J Matern Fetal Neonatal Med.

[32] Thummasorn S, Apaijai N, Kerdphoo S, Shinlapawittayatorn K, Chattipakorn SC, Chattipakorn N (2016). Humanin exerts cardioprotection against cardiac ischemia/reperfusion injury through attenuation of mitochondrial dysfunction. Cardiovasc Ther.

[33] Zhao ST, Huang XT, Zhang C, Ke Y (2012). Humanin protects cortical neurons from ischemia and reperfusion injury by the increased activity of superoxide dismutase. Neurochem Res.

[34] Kuliawat R, Klein L, Gong Z, Nicoletta-Gentile M, Nemkal A, Cui L, Bastie C, Su K, Huffman D, Surana M, Barzilai N, Fleischer N, Muzumdar R (2013). Potent humanin analog increases glucose-stimulated insulin secretion through enhanced metabolism in the beta cell. FASEB J.

[35] Zhang X, Urbieta-Caceres VH, Eirin A, Bell CC, Crane JA, Tang H, Jordan KL, Oh YK, Zhu XY, Korsmo MJ, Bachar AR, Cohen P, Lerman A, Lerman LO (2012). Humanin prevents intra-renal microvascular remodeling and inflammation in hypercholesterolemic ApoE deficient mice. Life Sci.

[36] Oh YK, Bachar AR, Zacharias DG, Kim SG, Wan J, Cobb LJ, Lerman LO, Cohen P, Lerman A (2011). Humanin preserves endothelial function and prevents atherosclerotic plaque progression in hypercholesterolemic ApoE deficient mice. Atherosclerosis.

[37] Black W, Chen Y, Matsumoto A, Thompson DC, Lassen N, Pappa A, Vasiliou V (2012). Molecular mechanisms of ALDH3A1-mediated cellular protection against 4-hydroxy-2-nonenal. Free Radic Biol Med.

[38] Estey T, Piatigorsky J, Lassen N, Vasiliou V (2007). ALDH3A1: a corneal crystallin with diverse functions. Exp Eye Res.

[39] Voulgaridou GP, Tsochantaridis I, Mantso T, Franco R, Panayiotidis MI, Pappa A (2017). Human aldehyde dehydrogenase 3A1 (ALDH3A1) exhibits chaperone-like function. Int J Biochem Cell Biol.

[40] Frederick Vuvor HM, Ndanu T, Harrison O (2017). Effect of lysine supplementation on hypertensive men and women in selected peri-urban community in Ghana. BMC Nutrition.

[41] Flodin NW (1997). The metabolic roles, pharmacology, and toxicology of lysine. J Am Coll Nutr.

[42] Cabre A, Lazaro I, Girona J, Manzanares J, Marimon F, Plana N, Heras M, Masana L (2007). Retinol-binding protein 4 as a plasma biomarker of renal dysfunction and cardiovascular disease in type 2 diabetes. J Intern Med.

